# Fenugreek Sprouts Around the World: Exploring Therapeutic and Nutritional Benefits

**DOI:** 10.1002/fsn3.4668

**Published:** 2024-12-15

**Authors:** Furkan Çoban

**Affiliations:** ^1^ Department of Plant Breeding The Swedish University of Agricultural Sciences Lomma Sweden; ^2^ Department of Field Crops, Faculty of Agriculture Atatürk University Erzurum Türkiye

**Keywords:** anti‐diabetic, antioxidant, fenugreek, genotype, mineral, sprouts

## Abstract

This study investigates the therapeutic and nutritional potential of fenugreek sprouts from 30 diverse genotypes sourced from various regions. The aim was to characterize and compare their therapeutic attributes, including antioxidant capacity, antidiabetic, and anti‐cholinesterase activities, along with their nutritional compositions, particularly minerals, and protein content. Results revealed significant variations among the genotypes in terms of their therapeutic properties. China genotypes exhibited notable α‐amylase inhibition 64.57%, suggesting potential antidiabetic properties, while South Sudan genotypes demonstrated significant acetylcholinesterase (14.44%) and butyrylcholinesterase inhibitions, indicating possible cognitive health benefits. The Morocco and Konya/Türkiye genotypes exhibited noteworthy antioxidant effects, with showing DPPH^**•**^ scavenging activities of 7.79% and 7.23%, and ABTS^**•**+^ activities of 27.87% and 27.31%, respectively. Mineral analysis revealed considerable differences across genotypes. Israel genotypes had the highest iron content (43.18 mg/100 g), Sivas/Türkiye genotype had the highest potassium levels (2259.87 mg/100 g), and Kayseri/Türkiye genotype had the highest sodium content (616.91 mg/100 g). Ukraine genotypes contained the most magnesium (266.61 mg/100 g), while Israel genotypes also had the highest zinc content (54.44 mg/100 g). The protein content of the fenugreek sprouts varied significantly, with Corum/Türkiye showing the highest protein content at 5.75/100 g. Principal component analysis (PCA) highlighted the relationships among the mineral nutrients and protein content, revealing distinct groupings of genotypes based on their mineral compositions. Correlation analysis further elucidated the associations between various minerals and protein content. In conclusion, this study underscores the potential therapeutic and nutritional significance of fenugreek sprouts.

## Graphical abstract

This study delves into the therapeutic and nutritional qualities of fenugreek sprouts sourced from 30 diverse genotypes across different regions. Findings unveil significant genotype‐based variations in therapeutic attributes, including noteworthy antidiabetic and cognitive health benefits among specific genotypes. Moreover, mineral composition analysis highlights distinct groupings based on mineral compositions, emphasizing the potential therapeutic, and nutritional significance of fenugreek sprouts.

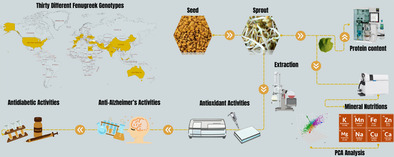

## 1 Introduction

The rapid growth of the world's population and the preservation of biodiversity are among the fundamental challenges of the near future. These challenges are primarily directed toward the agricultural sector, a continuously evolving field that meets basic human needs. The importance of agriculture in tackling such global issues cannot be overstated, as innovations and sustainable solutions within this sector can significantly enhance the quality of life. Plant cultivation is particularly crucial in representing biodiversity, demonstrated by the rich variety of plant species, products, and bioprocesses (Frison, Cherfas, and Hodgkin 2011; Allen and Prosperi 2016; Çakmakçı, Salık, and Çakmakçı 2023).

Among these bioprocesses, germination is widely used in the food industry, economically enhance the nutritional value of seeds and grains. Through germination, macronutrients are broken down into more absorbable forms, making sprouts nutritionally superior by improving their nutrient profiles and reducing anti‐nutritional components. This process enhances both digestibility and sensory qualities. Sprouts are rich in phenolic compounds, vitamins, and minerals, which play a critical role in promoting human nutrition and conserving biodiversity (Liu et al. 2017; Zhang et al. 2020). Sprouted seeds of fenugreek offer abundant health‐promoting phytochemicals, notably minerals and antioxidants (Khan, Wu, and Dolzhenko 2018; Wani and Kumar 2018; Pająk et al. 2019; Ebert 2022; Eswaranpillai, Murugesan, and Karuppiah 2023; Sura et al. 2023). With their recognized role as functional foods, sprout consumption has surged in recent years, prompting studies that examine how sprouting affects nutritional content, phytochemical profiles, and potential biological benefits (Benincasa et al. 2019; García et al. 2023).

Fenugreek ( *Trigonella foenum‐graecum* L.), an annual leguminous plant of the Fabaceae family, is widely consumed globally. Originating from the Indian subcontinent and the Eastern Mediterranean, fenugreek is among the oldest known medicinal plants and is valued in these regions as a food, spice, and traditional medicine (Acharya, Thomas, and Basu 2006; Alu'datt et al. 2024). Fenugreek seeds offer an abundance of nutrients and bioactive compounds, and research highlights their extensive health benefits, including anti‐inflammatory (Liu, Kakani, and Nair 2012), anti‐cancer (Shabbeer et al. 2009; Alsemari et al. 2014), antioxidant (Naidu et al. 2011) properties. Fenugreek seeds, leaves, and extracts have also demonstrated promising potential in managing diabetes, as shown in rat models where streptozotocin‐induced diabetes was treated with fenugreek seed extract (Nathiya, Janani, and Kannan 2020; Jin et al. 2020).

Furthermore, germinated fenugreek seeds exhibit higher antioxidant levels and improved antidiabetic properties compared to boiled seeds. This is attributed to the increased release or bioavailability of bound antioxidants during germination. The variations in antioxidant properties are influenced by environmental factors, genotype, and seed chemical composition (Khan, Wu, and Dolzhenko 2018; Wani and Kumar 2018). Studies have also pointed out the significant genetic variability among fenugreek plants (Maleki, Shojaeiyan, and Mokhtassi‐Bidgoli 2021; Maloo, Sharma, and Soan 2023; Shekhawat et al. 2023; Haliloğlu et al. 2024).

Sprouted seeds, including fenugreek, have been identified as a potential solution for nutritional security. With their rich nutritional profiles, they are considered an ideal crop for agronomic biofortification (Di Gioia et al. 2023). Given the increasing interest in healthy lifestyles and disease prevention, sprouts have gained popularity as highly sought‐after products. They are easy to produce and can be used in various culinary applications such as sandwiches, salads, soups, desserts, and beverages. Their delicate texture, vibrant colors, and high palatability further enhance their culinary value (Wojdyło et al. 2020; Choe, Yu, and Wang 2018).

The primary objectives of this study are as follows: (a) to assess the antioxidant capacity, anti‐diabetic, and anti‐cholinesterase activities of sprouted fenugreek seeds from 30 different genotypes, providing insights into their therapeutic benefits; (b) to determine the mineral composition and protein content of the sprouted seeds, exploring their potential as a functional food; and (c) to emphasize the importance of biodiversity by examining the genetic variability among fenugreek genotypes, showcasing how this diversity can contribute to both human health and sustainable agricultural practices. Ultimately, this study aims to contribute to the growing body of knowledge on the health‐promoting benefits of sprouted fenugreek seeds, while also highlighting their role in improving biodiversity and supporting nutrition security.

## 2 Materials and Methods

### 2.1 Plant Materials

**FIGURE 1:**
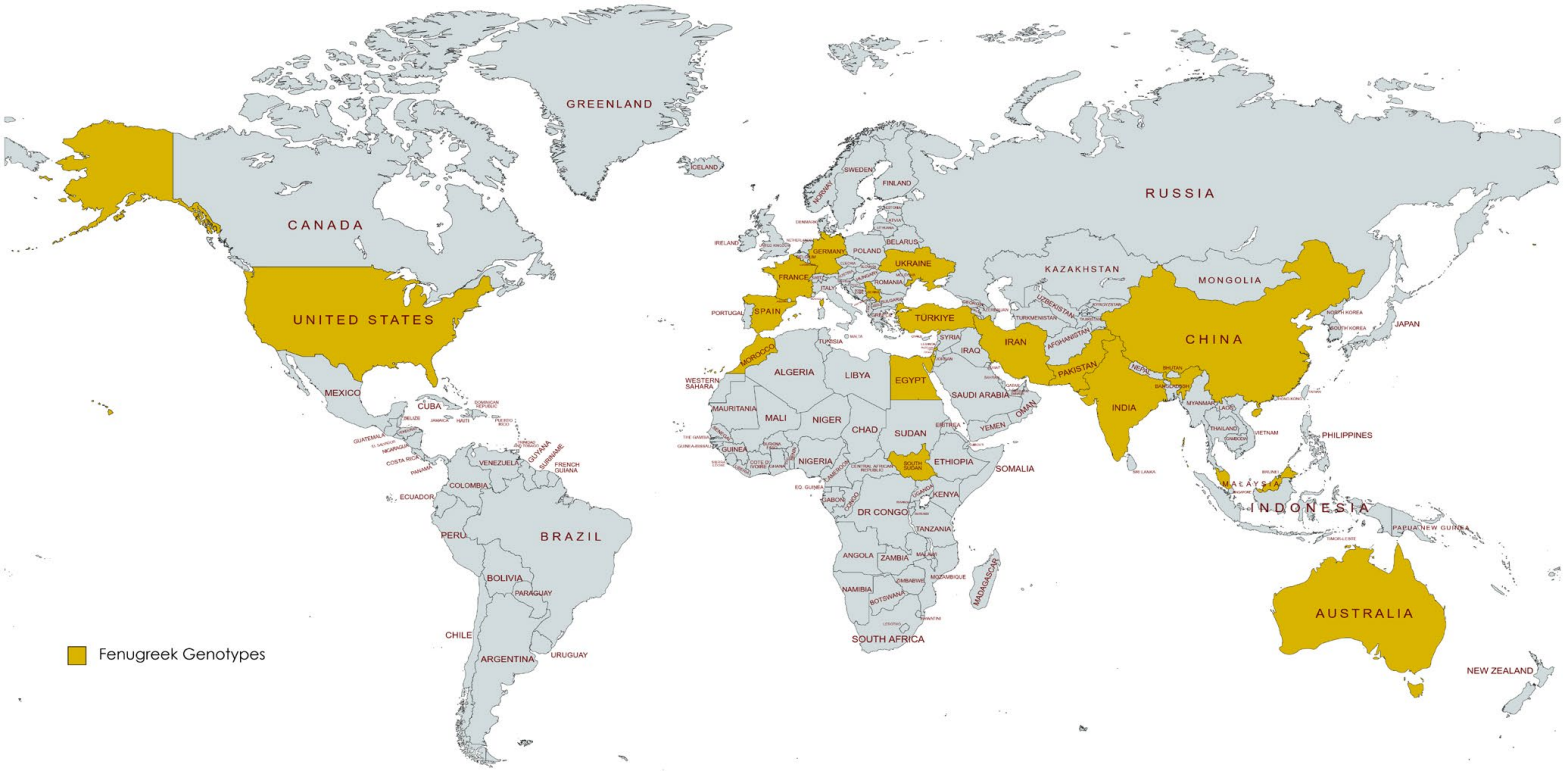
The countries from which the fenugreek seeds were sourced for sprout.

Thirty different genotypes were integrated into the research, and these genotypes were obtained from regions in Turkey that are important for fenugreek cultivation, as well as from other countries where it is cultivated. The genotypes from Turkey and Iran were sourced from local farmers engaged in fenugreek farming in the region. The other genotypes were obtained from research institutes, universities, and local vendors. Detailed information regarding the plant materials employed in this investigation, along with their specific origins, are outlined in Table 1, Figure 1.

**TABLE 1:** The genotype codes, origins, and accession numbers of fenugreek genotypes.

Genotype code	Accessions	Origin of genotype	Genotype Code	Accessions	Origin of genotype
G1	ZFTB0029	Ahvaz/Iran	G16	ZFTB0012	South Sudan
G2	ZFTB0004	Sanliurfa/Türkiye	G17	ZFTB0020	Selmas/Iran
G3	ZFTB0034	India	G18	ZFTB0015	Australia
G4	ZFTB0032	Sri Lanka	G19	ZFTB0018	Ukraine
G5	ZFTB0031	Pakistan	G20	ZFTB0036	USA
G6	ZFTB0009	Germany	G21	ZFTB0010	Kermanshah/Iran
G7	ZFTB0022	Serbia	G22	ZFTB0017	Morocco
G8	ZFTB0024	Tokat/Türkiye	G23	ZFTB0007	Egypt
G9	ZFTB0026	Malaysia	G24	ZFTB0023	Israel
G10	ZFTB0028	Karaman/Türkiye	G25	ZFTB0005	Sivas/Türkiye
G11	ZFTB0021	Kayseri/Türkiye	G26	ZFTB0019	China
G12	ZFTB0011	Berkem/Türkiye	G27	ZFTB0027	Konya/Türkiye
G13	ZFTB0013	Guraslan/Türkiye	G28	ZFTB0035	Ciftci/Türkiye
G14	ZFTB0016	Spain	G29	ZFTB0006	Corum/Türkiye
G15	ZFTB0003	Samsun/Türkiye	G30	ZFTB0014	France

### 2.2 Seed Germination

The germination procedure followed the methodology detailed in the publication by Pająk et al. (2019). Seeds were sterilized in 96% ethanol for 1 min. Subsequently, fenugreek genotype seeds were soaked in deionized water at a 1:10 (m/v) ratio for 4 h. Following the removal of the soaking water, the seeds were evenly distributed onto sterile, stackable trays and subjected to a twice‐daily, 30‐s rinsing regimen using deionized water to prevent seed decay. The germination procedure was iterated four times for every individual genotype. The germination process of fenugreek genotypes occurred within a controlled environment, maintained at approximately 22°C ± 2°C with a 12‐h day and night cycle. After 7 days of growth initiation, the sprouted seeds were harvested. The sprouts were lyophilized (Alpha 1‐2 LSCbasic, Germany), ground (IKA A11 basic Analytical mill 115V, IKA Germany), and stored in darkness for subsequent analyses.

### 2.3 Determination of Mineral Nutrients and Crude Protein

Fenugreek sprouts samples underwent dehydration in an oven at 68°C for 48 h. The nitrogen levels within the plant samples were determined by employing the Kjeldahl method (Bremner 1996). The calculation of crude protein (CP) involved multiplying the nitrogen content by a factor of 6.25, following the recommendation provided by Mariotti, Tomé, and Mirand (2008). Macro elements (P, K, Mg, Ca, and Na) and microelements (Fe, Mn, Cu, Zn, and B) were assessed after wet digestion of the dried and finely ground samples. The finely ground sprout samples underwent incineration via microwave treatment (Bergh of Speedwave Microwave Digestion Equipment MWS‐2) using the procedure according to U.S. EPA method 3052 (USEPA 1997). The quantification of macro and microelement content within the tissue was conducted using an Agilent 7800 ICP‐MS system (Agilent Technologies, Santa Clara, CA, USA).

### 2.4 Extraction

The fresh plant material from 30 fenugreek shoots (2 g) was first pulverized and then extracted with 20 mL of methanol using a mechanical mixer at 150 rpm at room temperature for 3 days and 8 h. After extraction, the solutions were filtered. The resulting filtrates were then concentrated using a rotary evaporator, and the extracts were weighed.

### 2.5 α‐Glucosidase Inhibition Assay

The assay for inhibiting the α‐glucosidase enzyme followed the procedure delineated by Praparatana et al. (2022), with adjustments made as per the modifications proposed by Yuca et al. (2021). In a 96‐well microplate, a blend was concocted by combining 50 μL of phosphate buffer (50 mM, pH 6.9), 10 μL of α‐glucosidase enzyme (1 Unit/mL), and 20 μL of samples (concentration range: 1‐5000 μg/mL). This amalgam underwent incubation at 37°C for 5 min. Subsequently, 20 μL of 3 mM p‐nitrophenyl‐α‐D‐glucopyranoside (pNPG) was introduced as the substrate, followed by another incubation at 37°C for 30 min. The reaction was brought to a halt by adding 50 μL of 0.1 M sodium carbonate (Na₂CO₃). All solutions were prepared within a buffered system. Acarbose served as the positive control. The quantification of the yellow p‐nitrophenol (pNP) produced was carried out at 405 nm. Each trial underwent replication three times. The resulting data were computed using the subsequent formula:

$$\%\text{Inhibition}=\left[\left(A_{\text{control}}-A_{\text{sample}}\right)/A_{\text{control}}\right]\times 100$$

### 2.6 α‐Amylase Inhibition Assay

The assay for inhibiting the α‐amylase enzyme followed the protocol outlined by Nampoothiri et al. (2011) with adjustments as per the modifications proposed by Yuca et al. (2021). In a microplate, 100 μL of the sample (concentration range: 1‐5000 μg/mL) was combined with a 1% starch solution in 20 mM sodium phosphate buffer (pH 6.9 with 6 mM sodium chloride) and incubated at 25°C for 10 min. Following this, 100 μL of pancreatic α‐amylase (0.5 mg/mL) was added to all wells. The samples underwent an additional incubation at 25°C for 10 min. Post reaction cessation, 200 μL of dinitrosalicylic acid (DNS) color reagent was introduced. The plates were incubated at 100°C for 5 min and then allowed to cool to room temperature. Subsequently, 50 μL from each tube was transferred to a 96‐well microplate, and the reaction mixture in each well was diluted by adding 200 μL of distilled water. The absorbances of the samples were measured at 540 nm. Acarbose served as the positive control. Each experiment was repeated three times. The resulting data were computed using the subsequent equation:

$$\%\text{Inhibition}=\left[\left(A_{\text{control}}-A_{\text{sample}}\right)/A_{\text{control}}\right]\times 100$$

### 2.7 Acetylcholinesterase (AChE) and Butyrylcholinesterase (BChE) Inhibition Assay

The assays for inhibiting AChE and BChE followed the procedure delineated by Ingkaninan et al. (2000) with minor adaptations as per the modifications proposed by Karakaya et al. (2023). In a 96‐well plate, a combination of 125 μL of 5,5′‐dithiobis‐(2‐nitrobenzoic acid) (3 mM, DTNB, Ellman's Reagent), 25 μL of the respective substrate (15 mM, acetylthiocholine iodide for AChE and butyrylthiocholine iodide for BChE), 50 μL of Tris–HCl buffer (50 mM, pH 8), and 25 μL of the sample was prepared. Following this, 25 μL of the corresponding enzyme (AChE and BChE) was added to the mixture, and the reaction underwent incubation for 10 min for the AChE inhibition assay and 15 min for the BChE inhibition assay. The reaction was then measured spectrophotometrically at 405 nm. Donepezil served as the positive control. Each assay was replicated three times. The percentage inhibition was determined using the subsequent equation:

$$\%\text{Inhibition}=\left[\left(A_{\text{control}}-A_{\text{sample}}\right)/A_{\text{control}}\right]\times 100$$

### 2.8 ABTS ^•+^ Scavenging Activity

The evaluation of ABTS^•+^ scavenging activity was carried out following the methodology introduced by Re et al. (1999). This approach assesses the test compound's capability to neutralize ABTS^•+^ radicals, generated through the oxidation of 2,2′‐azino‐bis(3‐ethylbenzothiazoline‐6‐sulfonic acid) (ABTS) with potassium persulfate. In this process, a 2 mM ABTS^•+^ solution served as the free radical, while α‐tocopherol and trolox were utilized as reference standards. The extracts were suitably diluted within the concentration range of 10–200 μg/mL. The antioxidant potential of the samples was gauged using the ABTS^•+^ solution. To establish a baseline, measurements were taken at 734 nm against a blank containing phosphate buffer (Karakaya et al. 2023). Each measurement was iterated three times to ensure precision and reliability. The calculation for ABTS^•+^ scavenging capacity was determined using the subsequent equation:

$$\%\text{Inhibition}=\left[\left(A_{\text{control}}-A_{\text{sample}}\right)/A_{\text{control}}\right]\times 100$$

A: absorbance value at 734 nm.

### 2.9 DPPH ^•^ Scavenging Activity

The evaluation of DPPH• scavenging activity was conducted following the methodology established by Blois (1958). This technique enables the assessment of the samples' capability to neutralize the DPPH• radical (1,1‐Diphenyl‐2‐picrylhydrazyl), indicating their potential as antioxidants. In this approach, a 1 mM DPPH• solution served as the free radical, and α‐tocopherol and trolox were utilized as reference standards. The extracts were suitably diluted within the concentration range of 10–200 μg/mL. The antioxidant efficacy of the samples was appraised using the DPPH• solution. To set a baseline, measurements were taken at 517 nm against a blank containing 99% ethanol (Karakaya et al. 2023). Each measurement was replicated three times to ensure precision and reliability. The calculation for DPPH• scavenging capacity was determined using the following equation:

$$\%\text{Inhibition}=\left[\left(A_{\text{control}}-A_{\text{sample}}\right)/A_{\text{control}}\right]\times 100$$

A: absorbance value at 517 nm.

### 2.10 Statistically Analysis

The trials of antioxidant capacity, anti‐diabetic, and anti‐cholinesterase activity were iterated three times, and statistical significance was assessed employing a Kruskal–Wallis analysis. Analysis of variance (ANOVA) and Duncan's multiple‐range test were conducted using JMP Pro 17 software to evaluate the mineral nutrition and protein content comprehensively. Additionally, the relationships among different genotypes were explored through principal component analysis (PCA), performed with the ggfortify package in RStudio, version 1.2.5042.

## 3 Results and Discussion

### 3.1 Extraction

**FIGURE 2:**
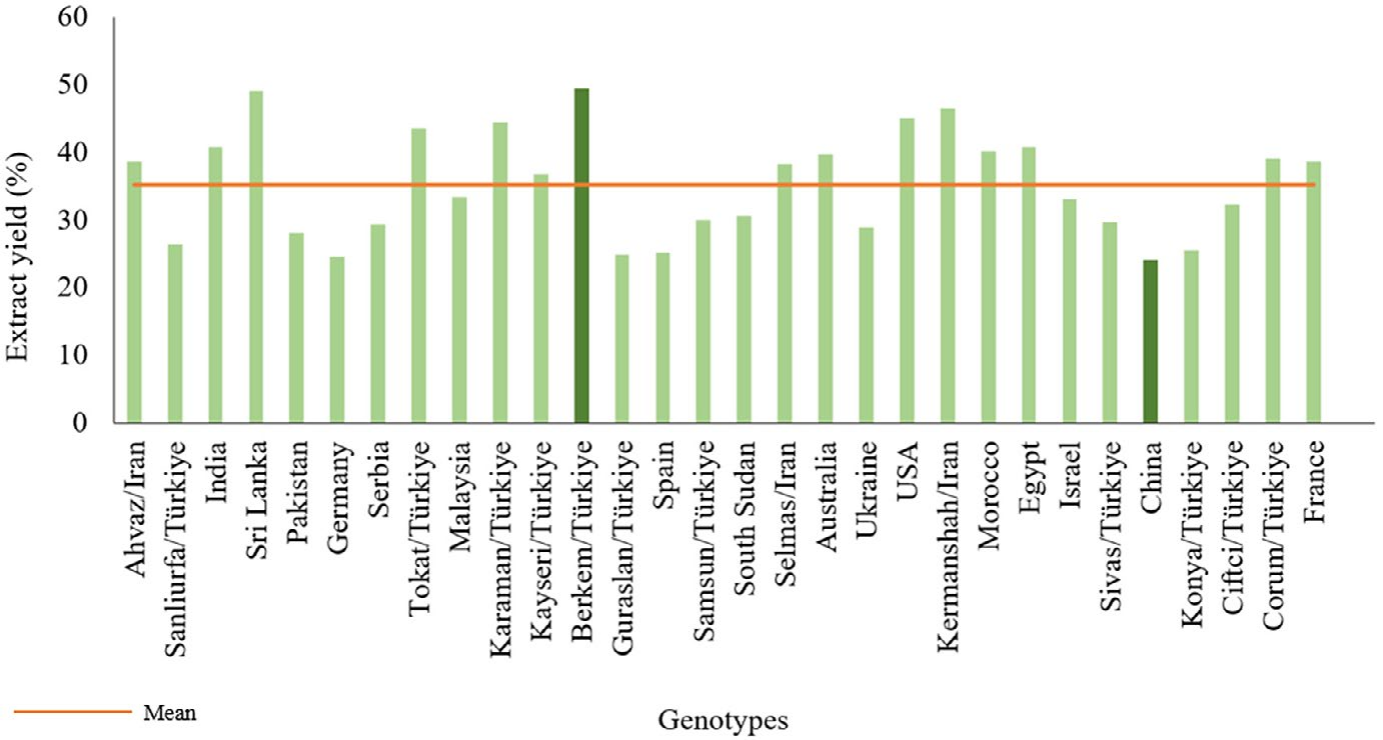
The extract yield of fenugreek sprouts.

Figure 2 displays the yields of the obtained extracts. Within the methanol extracts, Berkem/Türkiye (G_12_) exhibited the highest yield at 49.33%, while China (G_26_) showed the lowest yield at 24.06%. The average extract yield of different fenugreek sprouts was determined to be 35.19%. Additionally, extract yields obtained from all Iranian genotypes have been above the average.

### 3.2 Antidiabetic and Anticholinesterase Activities

Comparatively, G_30_ exhibited a lower α‐glucosidase inhibition (19.17%) at a concentration of 5000 μg/mL compared to the positive control, acarbose, which demonstrated a higher inhibition rate of 40.99% under the same conditions. Additionally, it is important to note that samples G_1_ to G_29_ did not show any significant α‐glucosidase inhibition at the specified concentration (Table 2).

**TABLE 2:** α‐Glucosidase and α‐amylase inhibitory activities of samples.

Genotype code	α‐Glucosidase inhibition (%) (at 5000 μg/mL)	α‐Amylase inhibition (%) (at 5000 μg/mL)
G1	N.D.	20.73 ± 0.52
G2	N.D.	18.55 ± 2.76
G3	N.D.	41.66 ± 1.95
G4	N.D.	41.57 ± 8.83
G5	N.D.	32.58 ± 14.39
G6	N.D.	33.02 ± 12.90
G7	N.D.	41.96 ± 10.64
G8	N.D.	44.71 ± 9.61
G9	N.D.	51.40 ± 11.21
G10	N.D.	47.35 ± 4.48
G11	N.D.	38.53 ± 9.55
G12	N.D.	41.29 ± 15.20
G13	N.D.	52.72 ± 10.91
G14	N.D.	40.99 ± 6.56
G15	N.D.	39.28 ± 9.28
G16	N.D.	32.77 ± 0.76
G17	N.D.	39.26 ± 1.52
G18	N.D.	27.66 ± 8.37
G19	N.D.	30.45 ± 2.95
G20	N.D.	34.15 ± 1.62
G21	N.D.	31.27 ± 5.38
G22	N.D.	46.35 ± 12.82
G23	N.D.	47.93 ± 0.44
G24	N.D.	50.36 ± 0.41
G25	N.D.	63.00 ± 2.55
G26	N.D.	64.57 ± 0.82
G27	N.D.	30.91 ± 0.51
G28	N.D.	33.15 ± 0.86
G29	N.D.	47.86 ± 1.70
G30	19.17 ± 8.65	49.92 ± 2.12
Acarbose	40.99 ± 2.55	80.40 ± 4.97

Based on the provided data for α‐amylase inhibition at a concentration of 5000 μg/mL, sample G_26_ exhibited the highest inhibition (64.57%), followed by G_25_ (63.00%), G_13_ (52.72%), and G_9_ (51.40%). On the other hand, G_2_ showed the lowest α‐amylase inhibition (18.55%). Comparatively, acarbose demonstrated the highest α‐amylase inhibition (80.40%) among all samples (Table 2).

In a study, on the fourth day of germination, fenugreek sprouts of the IM6 genotype were transformed into aqueous extract lyophilized powder, denoted as IM6E. Specifically, under in vitro conditions, IM6E treatment demonstrated a 41.64% inhibition of α‐amylase activity. Notably, the α‐glucosidase inhibition activity was exceptionally strong at 95.24% (Laila et al. 2023). A study aimed to assess how the duration of sprouting in fenugreek seeds over a 10‐day period affects their α‐amylase inhibitory activity. The results showed that the α‐amylase inhibitory activity of fenugreek seeds was affected by the germination process, with the highest inhibition percentage observed on the third day of sprouting (38.2%) for the aqueous extract. Acarbose exhibited a 31.7% inhibition of amylase. An IC_50_ assay of the fenugreek extract from the third day yielded a value of 19.87 mg/mL, whereas for acarbose, the IC_50_ value obtained was 4.33 mg/mL (Banerjee and Jain 2022).

In terms of AChE inhibition at a concentration of 100 μg/mL, several samples were compared, excluding Donepezil, which demonstrated the highest inhibition at 99.85%. Among these samples, G_16_ exhibited the most significant inhibition, with an inhibition rate of 14.44%. Following closely, G_24_ displayed an inhibition rate of 14.03%, and G_21_ demonstrated an inhibition of 12.64%. On the other end of the spectrum, indicating the lowest effective inhibitory activity, were sample G_28_ exhibited a slightly lower inhibitory effect, with an inhibition rate of 1.88%. Additionally, G_25_, G_26_, and G_27_ showed no inhibitory activity against AChE (Table 3).

**TABLE 3:** Anticholinesterase inhibitory activities of samples.

Genotype code	Acetylcholinesterase inhibition (%) (at 100 μg/mL)	Butyrylcholinesterase inhibition (%) (at 500 μg/mL)
G1	2.41 ± 3.25	N.D.
G2	4.35 ± 1.12	4.11 ± 3.05
G3	4.40 ± 0.91	N.D.
G4	6.69 ± 0.60	6.87 ± 5.30
G5	6.91 ± 1.29	1.23 ± 2.10
G6	9.36 ± 1.44	N.D.
G7	9.92 ± 1.04	4.21 ± 3.30
G8	11.28 ± 0.71	2.63 ± 4.68
G9	12.22 ± 1.74	N.D.
G10	6.72 ± 0.60	8.17 ± 1.43
G11	9.59 ± 2.25	8.23 ± 0.95
G12	11.21 ± 0.20	6.59 ± 5.35
G13	11.91 ± 0.69	N.D.
G14	12.52 ± 0.22	0.47 ± 7.40
G15	12.21 ± 2.86	3.37 ± 4.08
G16	14.44 ± 0.62	12.96 ± 2.40
G17	5.60 ± 7.03	N.D.
G18	6.30 ± 1.76	0.33 ± 2.36
G19	8.87 ± 5.83	N.D.
G20	10.14 ± 2.81	1.08 ± 3.50
G21	12.64 ± 5.23	3.82 ± 3.07
G22	11.63 ± 6.10	2.37 ± 2.79
G23	9.13 ± 5.64	4.86 ± 6.78
G24	14.03 ± 0.89	7.87 ± 2.41
G25	N.D.	N.D.
G26	N.D.	N.D.
G27	N.D.	2.37 ± 1.18
G28	1.88 ± 0.73	N.D.
G29	3.89 ± 2.24	N.D.
G30	2.48 ± 4.16	N.D.
Donepezil	99.85 ± 0.41	98.77 ± 0.49

Regarding the BChE inhibition at a concentration of 500 μg/mL, various samples were evaluated and compared with donepezil, which exhibited the highest inhibition at 98.77%. Among these, G_16_ demonstrated notable inhibition with an inhibition rate of 12.96%. Following closely was G_11_ with an inhibition rate of 8.23%. G_18_ showed a slightly lower inhibition at 0.33%. Donepezil displayed the most significant inhibitory effect on BChE among all the samples tested (Table 3).

A previous study aimed to evaluate the biological efficacy of *T. foenum‐graecum* , particularly its impact on α‐amylase and AChE inhibition. The results indicated that *T. foenum‐graecum* demonstrated significant α‐amylase inhibition activity, with inhibition percentages ranging from 9.43% to 24.95%. Additionally, the plant exhibited AChE inhibition potential, with inhibition ranging from 0.37% to 46.88% (Hafeez et al. 2023). As far as we are aware, our study represents the first investigation specifically focusing on the anticholinesterase effects of fenugreek sprouts.

In the context of diabetes, studies suggest that fenugreek sprout may play a role in managing blood sugar levels. It is believed to enhance insulin sensitivity and reduce glucose absorption in the digestive tract. Additionally, its hypoglycemic effects may contribute to improved glycemic control, making fenugreek sprout a subject of interest for individuals with diabetes. In relation to Alzheimers disease, fenugreek sprout has been explored for its neuroprotective properties. Oxidative stress is implicated in the progression of Alzheimers, and the antioxidant potential of fenugreek sprout may help mitigate such stress, potentially slowing down cognitive decline. While research is ongoing and more clinical evidence is needed, the preliminary findings suggest that fenugreek could be a valuable dietary addition for those managing diabetes and potentially beneficial for brain health in the context of Alzheimer's disease. As always, it is essential for individuals to consult with healthcare professionals before making significant changes to their diet or lifestyle, especially in the management of chronic conditions.

### 3.3 Antioxidant Activity Assay

#### 3.3.1 DPPH ^•^ And ABTS ^·+^ Scavenging Activity

Antioxidant activity of samples was presented in Table 4. In DPPH radical scavenging tests, when the % inhibition values of the standards (α‐tocopherol [TC], troloks [TR]) and extracts were compared at a concentration of 50 μg/mL, it was determined that especially G_22_ had a higher % inhibition value on DPPH^•^. However, the % inhibition values of all samples were found very close to each other. When the % inhibition values of standards and extracts in ABTS cation radical scavenging tests were compared at 50 μg/mL concentration, it was seen that the G_22_ and G_27_ came to the fore in terms of antioxidant effect. In this context, the results were found to be compatible with DPPH radical scavenging capacity tests. The antioxidant capacities of the extracts were found to be below average compared to the standards. During the germination of plant seeds, a complex process of biotransformation occurs, resulting in alterations to the quantities of pre‐existing antioxidant compounds and the generation of new radical scavengers (Wu, Song, and Huang 2011). Germination and sprouting represent aerobic processes that lead to an upsurge in the activity of reactive oxygen species (ROS) (Oracz and Karpiński 2016). These ROS may deplete certain radical scavengers that were initially present in the seeds. In response to oxidative stress, the seeds may subsequently synthesize additional radical scavengers, such as ascorbic acid (Dziki and Gawlik‐Dziki 2019). Therefore, antioxidants are an integral part of the overall nutritional profiles of seed sprouts.

**TABLE 4:** ABTS^•+^ and DPPH^•^ scavenging activity test results for 100 μg/mL.

Genotype code	DPPH^•^ scavenging activity (% inhibition of 50 μg/mL ± standard deviation)	ABTS^•+^ scavenging activity (% inhibition of 50 μg/mL ± standard deviation)
G1	4.51 ± 0.006	23.11 ± 0.018
G2	3.72 ± 0.006	21.87 ± 0.014
G3	5.05 ± 0.005	23.54 ± 0.009
G4	3.19 ± 0.017	20.98 ± 0.022
G5	3.11 ± 0.015	21.25 ± 0.012
G6	1.61 ± 0.005	17.27 ± 0.006
G7	1.45 ± 0.025	19.22 ± 0.017
G8	3.34 ± 0.031	21.89 ± 0.022
G9	5.13 ± 0.037	25.43 ± 0.004
G10	2.19 ± 0.012	19.47 ± 0.004
G11	6.08 ± 0.028	25.86 ± 0.018
G12	1.01 ± 0.008	14.78 ± 0.008
G13	3.32 ± 0.005	22.16 ± 0.010
G14	1.86 ± 0.011	15.46 ± 0.003
G15	4.65 ± 0.006	23.38 ± 0.007
G16	3.76 ± 0.003	22.91 ± 0.019
G17	6.67 ± 0.012	26.57 ± 0.011
G18	5.04 ± 0.023	23.74 ± 0.006
G19	4.95 ± 0.007	23.05 ± 0.019
G20	6.07 ± 0.020	24.76 ± 0.005
G21	1.42 ± 0.017	18.60 ± 0.002
G22	7.79 ± 0.034	27.87 ± 0.013
G23	5.51 ± 0.008	24.98 ± 0.029
G24	6.78 ± 0.004	26.48 ± 0.005
G25	2.21 ± 0.026	20.18 ± 0.014
G26	2.42 ± 0.007	19.88 ± 0.001
G27	7.23 ± 0.012	27.31 ± 0.012
G28	6.87 ± 0.004	26.96 ± 0.006
G29	2.91 ± 0.049	19.80 ± 0.014
G30	6.75 ± 0.012	26.09 ± 0.006
α‐Tocopherol	85.16 ± 0.002	90.11 ± 0.001
Trolox	91.00 ± 0.006	99.13 ± 0.002

### 3.4 Total Protein and Mineral Nutritions Content

**FIGURE 3:**
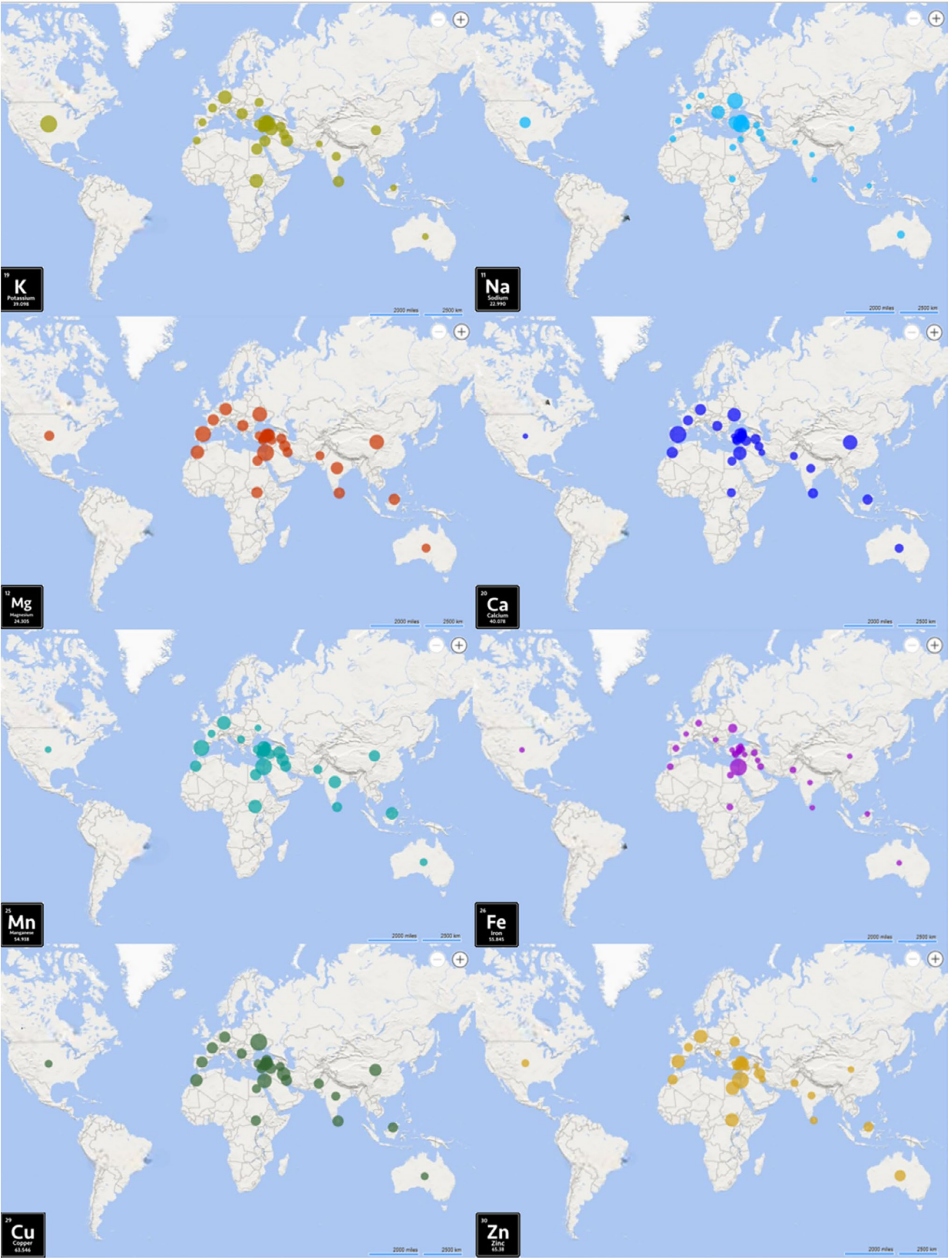
Maps of mineral nutrient profiles of fenugreek sprouts worldwide.

These essential minerals play vital roles in human nutrition. Each mineral plays a unique and indispensable role in supporting overall health and well‐being. Considering the significant role of these minerals, it is imperative to include high‐mineral foods in dietary regimen. It is also crucial to ensure adequate protein intake. Protein is essential for various physiological functions, including muscle repair and growth, enzyme production, and immune function. The mineral nutritions contents and protein content in different fenugreek genotype sprouts are presented in Table 5. The differences in mineral levels and protein content among different fenugreek genotype sprouts were found to be statistically significant (*p* < 0.001). Fenugreek genotype sprouts exhibited varying protein contents, with the highest levels observed in G_29_ (5.75/100 g) and G_17_ (5.73/100 g), and the lowest in G_21_ (2.62/100 g). The average protein content of the genotypes was found to be 3.98/100 g. In the Tukey post hoc analysis conducted (Table 5), it was observed that there is a high variation in mineral contents among genotypes and maps of mineral nutrient profiles of fenugreek sprouts worldwide are shown in Figure 3. In the sprouts of fenugreek, the mean values of genotypes were found to be 222.42 mg Na, 225.65 mg Mg, 1857.09 mg K, 100.48 mg Ca, 2.16 mg Mn, 11.88 mg Fe, 1.32 mg Cu, and 33.39 mg Zn per 100 g (Table 5). The highest level of Na, which is significant for maintaining electrolyte balance, fluid balance, nerve transmission, and muscle function, was determined in G_11_ (Kayseri, Türkiye) (616.91 mg 100 g). However, this genotype also had the lowest content of Mg (160.41 mg), Ca (49.29), and Mn (1.28). The values of calcium, which has an essential for bone and teeth health, as well as for muscle and nerve function, were found between 49.29 and 171.22 mg. The G_7_ (Spain) has stood out for its high calcium and manganese content (3.09 mg). Potassium (K) maintains fluid balance and supports nerve signals and muscle contractions, including those of the heart. The highest level of K was determined in G_25_ (Sivas/Türkiye), (2259.87 mg). The genotype G_27_ (Konya/Türkiye) exhibited the lowest potassium content and simultaneously had the lowest iron (7.30 mg) and copper content (0.68 mg). While the G_24_ (Israel) genotype stood out for its high iron content (43.18 mg), it also exhibited the highest zinc content (54.44). Iron is essential for oxygen transport and cellular energy production, while zinc is critical for immune response, wound healing, DNA synthesis, and growth. Finally, magnesium, crucial for energy production and muscle function, and copper, essential for red blood cell formation, connective tissue maintenance, and immune function, were noteworthy in the G14 (Ukraine) genotype, with content of 266.61 and 2.05 mg, respectively (Table 5). In general, it has been determined that fenugreek sprouts are a rich source of potassium, magnesium, and sodium and that there is significant variation among genotypes. Additionally, Pająk et al. (2019) have supported this statement, and our study has shown higher amounts of zinc, iron, potassium, and sodium compared to their study. According to Uwakiem (2021), an analysis of fenugreek sprouts nutritional value showed that iron (242 mg) levels were considerably higher, while potassium (469 mg) levels were lower when compared to this study. This difference is thought to result from genotype diversity, which plays a crucial role in determining the mineral composition of the sprouts. Dobrowolska‐Iwanek et al. (2022) noted that fenugreek sprouts are high in essential minerals like iron, magnesium, and calcium, which positions them as a valuable nutritional source for those aiming to increase their mineral consumption.

**TABLE 5:** Mineral and protein content of the sprouts from the 30 fenugreek genotypes.

Genotype code	Na (mg 100 g^−1^)	Mg (mg 100 g^−1^)	K (mg 100 g^−1^)	Ca (mg 100 g^−1^)	Mn (mg 100 g^−1^)	Fe (mg 100 g^−1^)	Cu (mg 100 g^−1^)	Zn (mg 100 g^−1^)	Protein content (g/100 g)
G1	115.51 ± 6.85 m‐p^a^	213.88 ± 6.63 f‐ı	2108.02 ± 96.50 bcd	71.70 ± 2.82 L	2.25 ± 0.09 e‐h	11.41 ± 0.18 hıj	1.42 ± 0.03 e‐h	25.61 ± 0.68 ıj	4.58 ± 0.10 de
G2	135.63 ± 7.75 j‐o^b^	199.64 ± 8.40 h‐k	2132.94 ± 43.18 bcd	99.80 ± 1.66 f‐ı	2.06 ± 0.07 g‐j	9.29 ± 0.17 nop	1.36 ± 0.03 f‐j	25.50 ± 0.67 ıj	4.61 ± 0.09 de
G3	124.61 ± 9.01 k‐p	237.60 ± 12.16 c‐h	1698.19 ± 33.20 g‐l	97.57 ± 1.23 g‐j	2.45 ± 0.17 b‐e	10.80 ± 0.29 klm	1.20 ± 0.03 lm	29.89 ± 0.98 g	4.93 ± 0.06 c
G4	87.93 ± 7.36 p	232.72 ± 7.81 d‐h	1933.16 ± 69.54 c‐h	104.31 ± 2.34 fgh	2.17 ± 0.05 e‐h	10.39 ± 0.19 lm	1.39 ± 0.05 f‐ı	28.43 ± 0.33 gh	4.65 ± 0.05 d
G5	94.90 ± 3.49 op	210.22 ± 7.20 f‐ı	1547.14 ± 36.39 j‐m	80.69 ± 2.59 kL	2.03 ± 0.11 g‐j	11.14 ± 0.15 ıjk	1.28 ± 0.02 h‐l	27.69 ± 0.31 ghı	4.65 ± 0.06 d
G6	161.66 ± 6.89 h‐m	244.47 ± 5.58 b‐f	2185.87 ± 101.18 abc	111.00 ± 4.55 c‐f	2.66 ± 0.08 bc	11.39 ± 0.15 hıj	1.38 ± 0.03 f‐j	45.38 ± 0.85 b	3.89 ± 0.05 g
G7	488.37 ± 13.26 b	236.58 ± 2.92 c‐h	1962.87 ± 54.28 b‐h	106.46 ± 3.99 efg	1.70 ± 0.09 kL	9.09 ± 0.10 nop	1.26 ± 0.02 ı‐l	22.29 ± 0.69 k‐n	2.84 ± 0.06 lm
G8	105.81 ± 5.99 nop	226.97 ± 3.44 e‐h	2060.22 ± 55.44 b‐e	95.14 ± 1.64 g‐j	2.12 ± 0.04 f‐ı	9.65 ± 0.12 n	1.34 ± 0.04 g‐k	24.48 ± 0.67 jk	3.69 ± 0.16 ghı
G9	88.35 ± 4.31 p	229.16 ± 6.13 d‐h	1504.18 ± 31.25 klm	103.49 ± 1.78 fgh	2.57 ± 0.08 bcd	10.24 ± 0.15 m	1.28 ± 0.03 h‐l	34.08 ± 1.54 f	4.44 ± 0.15 def
G10	283.02 ± 6.66 e	199.76 ± 5.42 hıj	1648.65 ± 27.40 h‐l	94.14 ± 1.64 hıj	2.02 ± 0.05 g‐j	11.79 ± 0.11 fgh	1.29 ± 0.02 g‐l	21.75 ± 0.15 lmn	3.83 ± 0.02 ghı
G11	616.91 ± 17.65 a	160.41 ± 5.85 k	1597.68 ± 31.27 ı‐m	49.29 ± 1.78 m	1.28 ± 0.06 n	8.78 ± 0.12 p	1.11 ± 0.04 mno	22.40 ± 0.11 k‐n	4.47 ± 0.11 def
G12	252.97 ± 12.74 ef	211.85 ± 10.47 f‐ı	2137.47 ± 67.00 bcd	89.82 ± 1.64 ıjk	2.33 ± 0.07 d‐g	12.35 ± 0.09 def	1.16 ± 0.02 lmn	46.29 ± 1.03 b	3.88 ± 0.07 gh
G13	118.68 ± 9.56 L‐p	240.45 ± 10.06 b‐g	1822.78 ± 53.07 d‐k	119.31 ± 3.07 cd	2.45 ± 0.14 b‐e	12.56 ± 0.17 d	1.43 ± 0.04 efg	27.08 ± 0.73 hı	4.47 ± 0.04 def
G14	181.33 ± 6.56 g‐j	278.73 ± 5.88 ab	1644.10 ± 45.26 h‐l	171.22 ± 3.02 a	3.09 ± 0.06 a	11.91 ± 0.04 e‐h	1.42 ± 0.03 efg	45.49 ± 0.91 b	4.29 ± 0.04 f
G15	145.10 ± 5.21 ı‐n	168.84 ± 4.26 jk	1820.55 ± 48.05 d‐k	86.01 ± 3.84 jk	1.75 ± 0.07 jkl	7.44 ± 0.08 q	1.27 ± 0.02 ı‐l	21.09 ± 1.07 mn	3.23 ± 0.02 jk
G16	182.87 ± 5.31 ghı	234.86 ± 9.49 d‐h	2135.65 ± 47.75 bcd	88.44 ± 2.61 ıjk	2.76 ± 0.17 b	13.22 ± 0.14 c	1.25 ± 0.04 jkl	47.01 ± 1.17 b	3.33 ± 0.06 j
G17	168.82 ± 3.99 h‐k	221.69 ± 6.83 e‐h	1838.62 ± 30.75 d‐j	107.06 ± 3.62 d‐g	2.44 ± 0.14 c‐f	12.43 ± 0.09 de	1.22 ± 0.02 klm	23.06 ± 0.40 j‐m	5.39 ± 0.05 b
G18	224.64 ± 8.85 fg	199.64 ± 5.81 h‐k	1496.39 ± 52.88 lm	95.46 ± 3.17 g‐j	1.70 ± 0.09 kl	10.37 ± 0.06 lm	0.98 ± 0.02 o	38.91 ± 0.47 cd	3.62 ± 0.02 ı
G19	616.28 ± 15.76 a	266.61 ± 9.68 a‐d	1747.26 ± 51.65 e‐l	146.00 ± 3.82 b	1.65 ± 0.03 lm	20.38 ± 0.22 b	2.05 ± 0.04 a	33.79 ± 0.37 f	3.33 ± 0.06 j
G20	394.43 ± 9.04 cd	213.98 ± 8.34 f‐ı	2469.18 ± 75.65 a	56.36 ± 3.13 m	1.53 ± 0.02 lmn	9.51 ± 0.05 no	1.02 ± 0.02 no	29.29 ± 0.29 gh	3.65 ± 0.10 hı
G21	245.34 ± 8.85 ef	237.06 ± 4.03 c‐h	1882.23 ± 26.66 c‐ı	89.19 ± 3.74 ıjk	2.46 ± 0.03 b‐e	8.97 ± 0.09 op	1.67 ± 0.03 c	39.81 ± 0.35 c	2.62 ± 0.02 m
G22	164.91 ± 5.70 h‐l	254.14 ± 5.83 a‐e	1598.03 ± 52.63 ı‐m	120.59 ± 3.14 c	2.31 ± 0.05 d‐h	12.05 ± 0.07 d‐g	1.60 ± 0.03 cd	36.96 ± 0.08 de	3.33 ± 0.04 j
G23	149.14 ± 4.81 h‐n	224.00 ± 5.90 e‐h	1976.90 ± 52.25 b‐g	88.15 ± 4.34 ıjk	2.23 ± 0.07 e‐h	11.09 ± 0.16 jk	1.19 ± 0.02 lm	47.37 ± 0.31 b	2.77 ± 0.11 m
G24	190.49 ± 7.16 ghı	288.82 ± 2.57 a	1901.57 ± 29.33 c‐ı	146.37 ± 3.99 b	3.15 ± 0.04 a	43.18 ± 0.21 a	1.82 ± 0.02 b	54.44 ± 1.27 a	2.73 ± 0.09 m
G25	193.74 ± 5.73 gh	241.07 ± 7.79 b‐g	2259.57 ± 86.36 ab	79.96 ± 2.74 kl	1.62 ± 0.14 lm	11.68 ± 0.04 ghı	1.50 ± 0.04 def	33.98 ± 0.25 f	4.61 ± 0.07 de
G26	126.50 ± 7.17 k‐p	274.72 ± 6.94 abc	1879.78 ± 88.40 c‐ı	155.88 ± 4.29 b	2.38 ± 0.05 c‐f	10.85 ± 0.15 jkl	1.55 ± 0.04 cde	23.91 ± 0.24 jkl	3.87 ± 0.04 gh
G27	111.01 ± 5.51 nop	177.31 ± 8.13 ıjk	1319.81 ± 25.64 m	80.86 ± 2.17 kL	1.34 ± 0.03 mn	7.30 ± 0.09 q	0.68 ± 0.02 p	27.62 ± 0.31 ghı	4.43 ± 0.02 def
G28	428.06 ± 11.12 c	211.46 ± 8.32 f‐ı	1705.94 ± 39.73 f‐l	55.86 ± 2.35 m	2.00 ± 0.06 h‐k	7.36 ± 0.11 q	1.21 ± 0.05 klm	19.89 ± 0.11 n	4.40 ± 0.09 ef
G29	355.78 ± 9.76 d	204.36 ± 6.07 g‐j	2021.85 ± 28.46 b‐f	118.38 ± 3.71 cde	2.57 ± 0.16 bcd	10.56 ± 0.12 klm	0.97 ± 0.03 o	34.70 ± 0.67 ef	5.75 ± 0.09 a
G30	119.97 ± 5.71 L‐p	228.67 ± 4.98 d‐h	1676.16 ± 36.33 g‐l	105.89 ± 2.32 fgh	1.83 ± 0.07 ı‐l	9.09 ± 0.11 nop	1.39 ± 0.04 f‐ı	33.43 ± 0.37 f	3.01 ± 0.07 kL
Mean	222.42 ± 9.66	225.65 ± 8.33	1857.09 ± 54.90	100.48 ± 2.95	2.16 ± 0.08	11.88 ± 0.13	1.32 ± 0.03	32.39 ± 0.54	3.98 ± 0.07

^a^ Mean ± standard deviation.

^b^ Means in the same raw with the same letters are not significantly different (*p* < 0.05).

Additionally, sprouts have limited mineral intake during the early stages of seed germination. They are typically grown for a shorter period and cultivated in water or a moist environment. Therefore, the mineral content of sprouts may be lower compared to microgreens (Ebert 2022). Based on Khoja et al. (2020) analysis of fenugreek microgreen mineral content, fenugreek sprouts were found to be high in iron and zinc; however, the calcium content in microgreens was nearly seven times higher than that in sprout.

The activation of the enzyme phytase during sprouting is an important biochemical mechanism that breaks down phytates (phytic acid) present in seeds, increasing the bioavailability of minerals such as iron, zinc, and magnesium. Phytates are antinutrients that bind to minerals, hindering their solubility and absorption. However, during the sprouting process, the phytase enzyme becomes active, breaking down phytates and releasing minerals, thereby enhancing their absorption in the intestines. This process can increase the bioavailability of minerals, particularly iron and zinc, by 30%–50% (Afify et al. 2011; Hussain et al. 2022). Additionally, the increased enzymatic activity during sprouting (such as amylase and protease) helps break down complex compounds, further promoting the release of minerals (Gibson, Raboy, and King 2018; Budhwar, Sethi, and Chakraborty 2020). These biochemical changes explain the variation in mineral content and increased bioavailability in different genotypes of plants like fenugreek during the sprouting stage.

**FIGURE 4:**
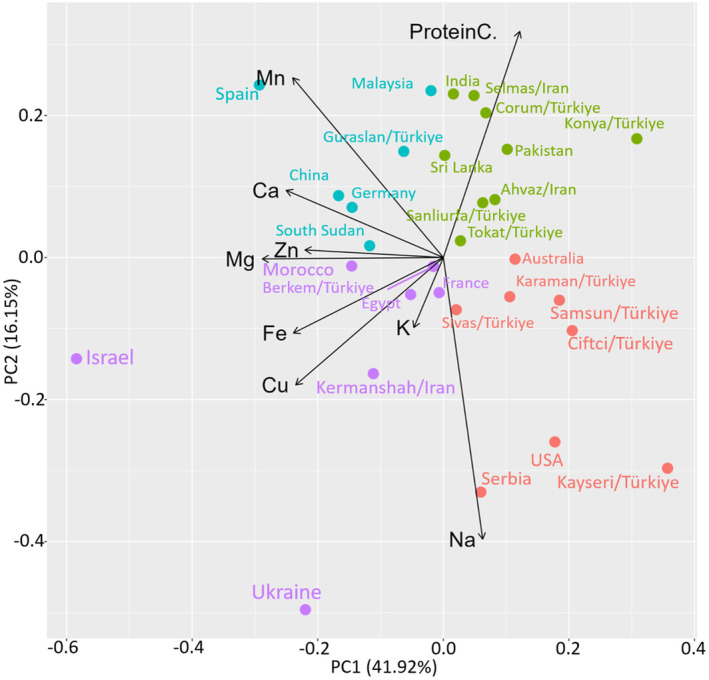
Biplot from principal‐component analysis (PCA) for mineral nutirions and protein content in sprouts from different fenugreek genotypes.

The mineral nutritions contents and protein content results were subjected to PCA. PCA is widely used to reduce the dimensionality of datasets, such as when analyzing mineral and protein content across different genotypes. By converting a large number of correlated variables into a smaller set of uncorrelated principal components, PCA helps highlight the primary sources of variation within the data. In the context of genotype analysis, PCA allows for a clearer understanding of differences in nutrient content among genotypes by simplifying complex data into its most significant factors (Kurita 2020; Li 2024). Two principal components (PC1 and PC2) with an Eigenvalues of > 1.0 explained 58.07% of the total variation (Table 6). Additional research indicates that the initial two components of a PCA ought to account for over 25% of the variance (Seymen 2021). The first principal component (PC1), explaining 41.92% of the total variation. Magnesium, calcium, manganese, iron, copper, and zinc have been the parameters most positively associated. PC2 has explained 16.14% of the variance, with Na and protein being the parameters most positively associated, while Mn has been the parameter most negatively associated. A loading plot was drawn using the PC1 and PC2 to display the relationship between the mineral nutritions and protein content (Figure 4). In this plot, angle < 90° between two vectors signifies a positive association, while an angle exceeding 90° indicates a negative relationship. Conversely, an angle close to or equal to 90° suggests the absence of a relationship (Lan et al. 2024). In this regard, there were significant positive correlation among Cu, Fe, Mg, Zn, Ca and Mn. Similarly, Na and protein content exhibited significant negative relationship. In the present study, loadings and score plots yielded significant results fort the mineral nutritions and protein content (Figure 4). There were four different sections in the score plot drawn using PC1 and PC2. The G_24_ (Israel) and G_19_ (Ukraine) genotypes, located in the positive region of both components, have been separated from other genotypes in terms of Na, Fe, and Cu contents. Table 7 provides an overview of the findings from the correlation analysis. Notably, a negative correlation emerged between protein content and all nutrients except Mn. Subsequent scrutiny of the correlation patterns within mineral nutrients revealed that Fe and Cu demonstrated a positive association with all minerals, whereas Mg and Zn displayed a positive correlation with other elements except Na. Furthermore, strong negative correlation was observed between Mn and Na.

**TABLE 6:** PCA results regarding mineral nutritions and protein content of different fenugreek genotypes sprout.

Items	PC1	PC2
Eigenvalue	3.77	1.45
Percentage of variance	41.92	16.14
Cumulative variance	41.92	58.07
Eigenvectors		
Na	−0.100	**0.639**
Mg	**0.464**	0.003
K	0.075	0.159
Ca	**0.403**	−0.152
Mn	**0.386**	**−0.407**
Fe	**0.384**	0.172
Cu	**0.378**	0.289
Zn	**0.355**	−0.017
Protein content	−0.196	**−0.513**

*Note:* The bold values indicate the variables that make the highest contribution to the respective principal components (PC1 or PC2).

**TABLE 7:** Correlation coefficients between mineral nutritions and protein content of different fenugreek genotypes sprout.

Parametreler	Na	Mg	K	Ca	Mn	Fe	Cu	Zn	Protein content
Na	1.000								
Mg	−0.137	1.000							
K	0.041	0.155	1.000						
Ca	−0.160	0.764	−0.133	1.000					
Mn	−0.420	0.624	0.130	0.579	1.000				
Fe	0.041	0.557	0.067	0.464	0.448	1.000			
Cu	0.064	0.718	0.125	0.543	0.333	0.545	1.000		
Zn	−0.156	0.489	0.168	0.360	0.572	0.523	0.229	1.000	
Protein content	−0.117	−0.273	−0.065	−0.112	0.028	−0.280	−0.384	−0.381	1.000

## 4 Conclusion

This study has revealed a remarkable diversity in the therapeutic and nutritional content of fenugreek sprouts across different genotypes. Therapeutic properties such as antidiabetic and antioxidant activities showed significant variations among genotypes. While some genotypes may play a significant role in diabetes treatment with their high α‐amylase inhibition, others have the potential to support cognitive health with their neuroprotective properties. At the same time, this study demonstrated that fenugreek sprouts are rich in essential minerals such as iron, zinc, and potassium, with certain genotypes standing out in terms of these mineral contents, highlighting the potential of fenugreek sprouts as a nutritious food source. Correlation and PCA analyses conducted in the study provided valuable insights into the relationships between different minerals and protein content, confirming that these variations among genotypes stem from genetic diversity. Future studies can build on these findings to offer more comprehensive evaluations on the use of fenugreek sprouts as functional foods and natural therapeutic agents.

## Author Contributions

**Furkan Çoban:** data curation (lead), formal analysis (lead), investigation (lead), methodology (lead), resources (lead), software (lead), validation (lead), writing – original draft (lead), writing – review and editing (lead).

## Data Availability

The data that support the findings of this study are available on request from the corresponding author upon reasonable request.

## References

[fsn34668-bib-0001] Acharya, S. , J. Thomas , and S. Basu . 2006. “Fenugreek: An "Old World" Crop for the "New World".” Biodiversity 7, no. 3–4: 27–30. 10.1080/14888386.2006.9712808.

[fsn34668-bib-0002] Afify, A. E. M. M. , H. S. El‐Beltagi , S. M. Abd El‐Salam , and A. A. Omran . 2011. “Bioavailability of Iron, Zinc, Phytate and Phytase Activity During Soaking and Germination of White Sorghum Varieties.” PLoS One 6, no. 10: e25512. 10.1371/journal.pone.0025512. 22003395 PMC3189212

[fsn34668-bib-0003] Allen, T. , and P. Prosperi . 2016. “Modeling Sustainable Food Systems.” Environmental Management 57: 956–975. 10.1007/s00267-016-0664-8. 26932834 PMC4828486

[fsn34668-bib-0004] Alsemari, A. , F. Alkhodairy , A. Aldakan , et al. 2014. “The Selective Cytotoxic Anti‐Cancer Properties and Proteomic Analysis of *Trigonella foenum‐graecum* .” BMC Complementary and Alternative Medicine 14: 1–9. 10.1186/1472-6882-14-114. 24679057 PMC4021494

[fsn34668-bib-0005] Alu'datt, M. H. , T. Rababah , S. Al‐ali , et al. 2024. “Current Perspectives on Fenugreek Bioactive Compounds and Their Potential Impact on Human Health: A Review of Recent Insights Into Functional Foods and Other High Value Applications.” Journal of Food Science 89: 1835–1864. 10.1111/1750-3841.16970. 38407443

[fsn34668-bib-0006] Banerjee, G. , and S. Jain . 2022. “Effect of Sprouting Time on the Alpha Amylase Inhibitory Activity of Fenugreek (*Trigonella Foenum graecum* L.) Seeds.” International Journal of Innovation and Multidisciplinary Research 2, no. 3: 35–38.

[fsn34668-bib-0007] Benincasa, P. , B. Falcinelli , S. Lutts , F. Stagnari , and A. Galieni . 2019. “Sprouted Grains: A Comprehensive Review.” Nutrients 11, no. 2: 421. 10.3390/nu11020421. 30781547 PMC6413227

[fsn34668-bib-0008] Blois, M. S. 1958. “Antioxidant Determinations by the Use of a Stable Free Radical.” Nature 181, no. 4617: 1199–1200.

[fsn34668-bib-0009] Bremner, J. M. 1996. “Nitrogen‐Total. Methods of Soil Analysis: Part 3 Chemical.” Methods 5: 1085–1121.

[fsn34668-bib-0010] Budhwar, S. , K. Sethi , and M. Chakraborty . 2020. “Efficacy of Germination and Probiotic Fermentation on Underutilized Cereal and Millet Grains.” Food Production, Processing and Nutrition 2: 12. 10.1186/s43014-020-00026-w.

[fsn34668-bib-0011] Çakmakçı, R. , M. A. Salık , and S. Çakmakçı . 2023. “Assessment and Principles of Environmentally Sustainable Food and Agriculture Systems.” Agriculture 13, no. 5: 1073. 10.3390/agriculture13051073.

[fsn34668-bib-0012] Choe, U. , L. L. Yu , and T. T. Wang . 2018. “The Science Behind Microgreens as an Exciting New Food for the 21st Century.” Journal of Agricultural and Food Chemistry 66, no. 44: 11519–11530. 10.1021/acs.jafc.8b03096. 30343573

[fsn34668-bib-0013] Di Gioia, F. , J. C. Hong , C. Pisani , S. A. Petropoulos , J. Bai , and E. N. Rosskopf . 2023. “Yield Performance, Mineral Profile, and Nitrate Content in a Selection of Seventeen Microgreen Species.” Frontiers in Plant Science 14: 1220691. 10.3389/fpls.2023.1220691. 37546245 PMC10399459

[fsn34668-bib-0014] Dobrowolska‐Iwanek, J. , P. Zagrodzki , A. Galanty , et al. 2022. “Determination of Essential Minerals and Trace Elements in Edible Sprouts From Different Botanical Families‐Application of Chemometric Analysis.” Food 11, no. 3: 371. 10.3390/foods11030371. PMC8834360 35159521

[fsn34668-bib-0015] Dziki, D. , and U. Gawlik‐Dziki . 2019. “Processing of Germinated Grains.” In Sprouted Grains, 69–90. Cambridge: AACC International Press. 10.1016/B978-0-12-811525-1.00004-X.

[fsn34668-bib-0016] Ebert, A. W. 2022. “Sprouts and Microgreens—Novel Food Sources for Healthy Diets.” Plants 11, no. 4: 571. 10.3390/plants11040571. 35214902 PMC8877763

[fsn34668-bib-0017] Eswaranpillai, U. , P. Murugesan , and P. Karuppiah . 2023. “Assess the Impact of Cultivation Substrates for Growing Sprouts and Microgreens of Selected Four Legumes and Two Grains and Evaluation of Its Nutritional Properties.” Plant Science Today 10, no. 2: 160–169. 10.14719/pst.2058.

[fsn34668-bib-0018] Frison, E. A. , J. Cherfas , and T. Hodgkin . 2011. “Agricultural Biodiversity Is Essential for Sustainable Improvement in Food and Nutrition Security.” Sustainability 3, no. 1: 238–253. 10.3390/su3010238.

[fsn34668-bib-0019] García, S. N. C. , R. Rodríguez‐Herrera , S. N. Flores , et al. 2023. “Sprouts as Probiotic Carriers: A New Trend to Improve Consumer Nutrition.” Food Chemistry: Molecular Sciences 7: 100185. 10.1016/j.fochms.2023.100185. 38155686 PMC10753383

[fsn34668-bib-0020] Gibson, R. S. , V. Raboy , and J. C. King . 2018. “Implications of Phytate in Plant‐Based Foods for Iron and Zinc Bioavailability, Setting Dietary Requirements, and Formulating Programs and Policies.” Nutrition Reviews 76, no. 11: 793–804. 10.1093/nutrit/nuy028. 30010865

[fsn34668-bib-0021] Hafeez, J. , M. Naeem , T. Ali , et al. 2023. “Comparative Study of Antioxidant, Antidiabetic, Cytotoxic Potentials, and Phytochemicals of Fenugreek ( *Trigonella foenum‐graecum* ) and Ginger ( *Zingiber officinale* ).” Journal of Chemistry 2023, no. 1: 3469727. 10.1155/2023/3469727.

[fsn34668-bib-0022] Haliloğlu, K. , H. Özer , S. Melik , F. Çoban , and A. Türkoğlu . 2024. “Exploring the Genetic Diversity and Population Structure of Fenugreek ( *Trigonella foenum‐graecum* L.) Genotypes Through Inter‐Primer Binding Site (iPBS)‐Retrotransposon Marker System.” Genetic Resources and Crop Evolution 71: 1–14. 10.1007/s10722-023-01849-5.

[fsn34668-bib-0023] Hussain, S. M. , S. Hanif , A. Sharif , F. Bashir , and H. M. Iqbal . 2022. “Unrevealing the Sources and Catalytic Functions of Phytase With Multipurpose Characteristics.” Catalysis Letters 1‐14: 1358–1371. 10.1007/s10562-021-03752-z.

[fsn34668-bib-0024] Ingkaninan, K. , C. M. De Best , R. Van Der Heijden , et al. 2000. “High‐Performance Liquid Chromatography With On‐Line Coupled UV, Mass Spectrometric and Biochemical Detection for Identification of Acetylcholinesterase Inhibitors From Natural Products.” Journal of Chromatography A 872, no. 1‐2: 61–73. 10.1016/S0021-9673(99)01292-3. 10749487

[fsn34668-bib-0025] Jin, C. , X. Miao , Y. Zhong , et al. 2020. “The Renoprotective Effect of Diosgenin on Aristolochic Acid I‐Induced Renal Injury in Rats: Impact on Apoptosis, Mitochondrial Dynamics and Autophagy.” Food & Function 11, no. 9: 7456–7467. 10.1039/D0FO00401D. 32789347

[fsn34668-bib-0026] Karakaya, S. , H. Yuca , G. Yılmaz , et al. 2023. “Phytochemical Screening, Biological Evaluation, Anatomical, and Morphological Investigation of *Ferula tingitana* L. (Apiaceae).” Protoplasma 260, no. 6: 1581–1601. 10.1007/s00709-023-01874-2. 37338647

[fsn34668-bib-0027] Khan, T. M. , D. B. C. Wu , and A. V. Dolzhenko . 2018. “Effectiveness of Fenugreek as a Galactagogue: A Network Meta‐Analysis.” Phytotherapy Research 2018, no. 32: 402–412. 10.1002/ptr.5972. 29193352

[fsn34668-bib-0028] Khoja, K. K. , A. F. Buckley , M. A. Aslam , P. Sharp , and G. O. Latunde‐Dada . 2020. “In Vitro Bioaccessibility and Bioavailability of Iron From Mature and Microgreen Fenugreek, Rocket and Broccoli.” Nutrients 12, no. 4: 1057. 10.3390/nu12041057. 32290311 PMC7231393

[fsn34668-bib-0029] Kurita, T. 2020. Principal Component Analysis (PCA): Computer Vision, 1–4. Cham, Switzerland: Springer. 10.1007/978-3-030-03243-2_649-1.

[fsn34668-bib-0030] Laila, O. , I. Murtaza , S. Muzamil , et al. 2023. “Enhancement of Nutraceutical and Anti‐Diabetic Potential of Fenugreek ( *Trigonella foenum‐graecum* ) Sprouts With Natural Elicitors.” Saudi Pharmaceutical Journal 31, no. 1: 1–13. 10.1016/j.jsps.2022.11.001. 36685305 PMC9845115

[fsn34668-bib-0031] Lan, Y. , R. Kuktaite , A. Chawade , and E. Johansson . 2024. “Chasing High and Stable Wheat Grain Mineral Content: Mining Diverse Spring Genotypes Under Induced Drought Stress.” PLoS One 19, no. 2: e0298350. 10.1371/journal.pone.0298350. 38359024 PMC10868752

[fsn34668-bib-0032] Li, H. 2024. “Principal Component Analysis.” In Machine Learning Methods. Singapore: Springer. 10.1007/978-981-99-3917-6_16.

[fsn34668-bib-0033] Liu, T. , G. G. Hou , M. Cardin , L. Marquart , and A. Dubat . 2017. “Quality Attributes of Whole‐Wheat Flour Tortillas With Sprouted Whole‐Wheat Flour Substitution.” LWT 77: 1–7. 10.1016/j.lwt.2016.11.017.

[fsn34668-bib-0034] Liu, Y. , R. Kakani , and M. G. Nair . 2012. “Compounds in Functional Food Fenugreek Spice Exhibit Anti‐Inflammatory and Antioxidant Activities.” Food Chemistry 131, no. 4: 1187–1192. 10.1016/j.foodchem.2011.09.102.

[fsn34668-bib-0035] Maleki, M. , A. Shojaeiyan , and A. Mokhtassi‐Bidgoli . 2021. “Genotypic Variation in Biochemical and Physiological Responses of Fenugreek ( *Trigonella foenum‐graecum* L.) Landraces to Prolonged Drought Stress and Subsequent Rewatering.” Scientia Horticulturae 287: 110224. 10.1016/j.scienta.2021.110224.

[fsn34668-bib-0036] Maloo, S. R. , R. Sharma , and H. Soan . 2023. “SSR Based Genetic Diversity Analysis in Fenugreek ( *Trigonella foenum‐graecum* L.) Genotypes.” Legume Research‐An International Journal 46, no. 3: 307–311. 10.18805/LR-4787.

[fsn34668-bib-0037] Mariotti, F. , D. Tomé , and P. P. Mirand . 2008. “Converting Nitrogen Into Protein‐Beyond 6.25 and Jones' Factors.” Critical Reviews in Food Science and Nutrition 48, no. 2: 177–184. 10.1080/10408390701279749. 18274971

[fsn34668-bib-0038] Naidu, M. M. , B. N. Shyamala , J. P. Naik , G. Sulochanamma , and P. Srinivas . 2011. “Chemical Composition and Antioxidant Activity of the Husk and Endosperm of Fenugreek Seeds.” LWT‐Food Science and Technology 44, no. 2: 451–456. 10.1016/j.lwt.2010.08.013.

[fsn34668-bib-0039] Nampoothiri, S. V. , A. Prathapan , O. L. Cherian , K. G. Raghu , V. V. Venugopalan , and A. Sundaresan . 2011. “In Vitro Antioxidant and Inhibitory Potential of *Terminalia Bellerica* and *Emblica officinalis* Fruits Against LDL Oxidation and Key Enzymes Linked to Type 2 Diabetes.” Food and Chemical Toxicology 49, no. 1: 125–131. 10.1016/j.fct.2010.10.006. 20951180

[fsn34668-bib-0040] Nathiya, S. , R. Janani , and V. R. Kannan . 2020. “Potential of Plant Growth Promoting Rhizobacteria to Overcome the Exposure of Pesticide in *Trigonella foenum‐graecum* (Fenugreek Leaves).” Biocatalysis and Agricultural Biotechnology 23: 101493. 10.1016/j.bcab.2020.101493.

[fsn34668-bib-0041] Oracz, K. , and S. Karpiński . 2016. “Phytohormones Signaling Pathways and ROS Involvement in Seed Germination.” Frontiers in Plant Science 7: 206808. 10.3389/fpls.2016.00864. PMC4908112 27379144

[fsn34668-bib-0042] Pająk, P. , R. Socha , J. Broniek , K. Królikowska , and T. Fortuna . 2019. “Antioxidant Properties, Phenolic and Mineral Composition of Germinated Chia, Golden Flax, Evening Primrose, Phacelia and Fenugreek.” Food Chemistry 275: 69–76. 10.1016/j.foodchem.2018.09.081. 30724250

[fsn34668-bib-0043] Praparatana, R. , P. Maliyam , L. R. Barrows , and P. Puttarak . 2022. “Flavonoids and Phenols, the Potential Anti‐Diabetic Compounds From Bauhinia Strychnifolia Craib Stem.” Molecules 27, no. 8: 2393. 10.3390/molecules27082393. 35458587 PMC9032570

[fsn34668-bib-0045] Re, R. , N. Pellegrini , A. Proteggente , A. Pannala , M. Yang , and C. Rice‐Evans . 1999. “Antioxidant Activity Applying an Improved ABTS Radical Cation Decolorization Assay.” Free Radical Biology and Medicine 26, no. 9–10: 1231–1237. 10381194 10.1016/s0891-5849(98)00315-3

[fsn34668-bib-0046] Seymen, M. 2021. “How Does the Flooding Stress Occurring in Different Harvest Times Affect the Morpho‐Physiological and Biochemical Characteristics of Spinach?” Scientia Horticulturae 275: 109713. 10.1016/j.scienta.2020.109713.

[fsn34668-bib-0047] Shabbeer, S. , M. Sobolewski , R. K. Anchoori , et al. 2009. “Fenugreek: A Naturally Occurring Edible Spice as an Anticancer Agent.” Cancer Biology & Therapy 8, no. 3: 272–278. 10.4161/cbt.8.3.7443. 19197146 PMC3095649

[fsn34668-bib-0048] Shekhawat, N. , V. S. Meena , K. Singh , K. Rani , and V. Gupta . 2023. “Studies on Genetic Variability, Heritability and Genetic Advance for Morphological Traits in Fenugreek ( *Trigonella foenum‐graecum* L.) for Arid Climate of Rajasthan. Legume Research‐An.” International Journal 46, no. 3: 312–315. 10.18805/LR-5046.

[fsn34668-bib-0049] Sura, S. , C. Kodikara , S. Acharya , A. Sabra , and C. Wijekoon . 2023. “Comparative Analysis of Bioactive Phenolic Compounds and Fatty Acids in Seeds and Seedlings of Canadian Alfalfa, Sainfoin, and Fenugreek.” Applied Biosciences 2, no. 3: 477–492. 10.3390/applbiosci2030030.

[fsn34668-bib-0050] USEPA . 1997. Method 3052: Microwave‐Assisted Acid Digestion of Siliceous and Organically Based Matrices. Washington, DC: USEPA.

[fsn34668-bib-0051] Uwakiem, M. K. 2021. “Growth and Productivity of Red Globe Grapevines as Affected With Spraying of Fenugreek Seed Sprout, Nano‐Boron and Moringa Extract.” Hortscience Journal of Suez Canal University 10, no. 1: 49–61. 10.21608/hjsc.2022.222804.

[fsn34668-bib-0052] Wani, S. A. , and P. Kumar . 2018. “Fenugreek: A Review on Its Nutraceutical Properties and Utilization in Various Food Products.” Journal of the Saudi Society of Agricultural Sciences 17, no. 2: 97–106. 10.1016/j.jssas.2016.01.007.

[fsn34668-bib-0053] Wojdyło, A. , P. Nowicka , K. Tkacz , and I. P. Turkiewicz . 2020. “Sprouts vs. Microgreens as Novel Functional Foods: Variation of Nutritional and Phytochemical Profiles and Their In Vitro Bioactive Properties.” Molecules 25, no. 20: 4648. 10.3390/molecules25204648. 33053861 PMC7587365

[fsn34668-bib-0054] Wu, Z. , L. Song , and D. Huang . 2011. “Food Grade Fungal Stress on Germinating Peanut Seeds Induced Phytoalexins and Enhanced Polyphenolic Antioxidants.” Journal of Agricultural and Food Chemistry 59, no. 11: 5993–6003. 10.1021/jf200776w. 21545178

[fsn34668-bib-0055] Yuca, H. , H. Özbek , L. Ö. Demirezer , H. G. Kasil , and Z. Güvenalp . 2021. “Trans‐Tiliroside: A Potent α‐Glucosidase Inhibitor From the Leaves of *Elaeagnus angustifolia* L.” Phytochemistry 188: 112795. 10.1016/j.phytochem.2021.112795. 34044297

[fsn34668-bib-0056] Zhang, X. , Z. Bian , X. Yuan , X. Chen , and C. Lu . 2020. “A Review on the Effects of Light‐Emitting Diode (LED) Light on the Nutrients of Sprouts and Microgreens.” Trends in Food Science & Technology 99: 203–216. 10.1016/j.tifs.2020.02.031.

